# A novel lncRNA, LUADT1, promotes lung adenocarcinoma proliferation via the epigenetic suppression of p27

**DOI:** 10.1038/cddis.2015.203

**Published:** 2015-08-20

**Authors:** M Qiu, Y Xu, J Wang, E Zhang, M Sun, Y Zheng, M Li, W Xia, D Feng, R Yin, L Xu

**Affiliations:** 1 Department of Thoracic Surgery, Nanjing Medical University Affiliated Cancer Hospital, Jiangsu Key Laboratory of Molecular and Translational Cancer Research, Cancer Institute of Jiangsu Province, Baiziting 42, Nanjing 210009, China; 2 The Fourth Clinical College of Nanjing Medical University, Nanjing, 210000, China; 3 The First Clinical College of Nanjing Medical University, Nanjing 210000, China; 4 Department of Scientific Research, Nanjing Medical University Affiliated Cancer Hospital, Cancer Institute of Jiangsu Province, Nanjing 210009, China; 5 Department of Biochemistry and Molecular Biology, Nanjing Medical University, Nanjing 210000, China; 6 Department of Nursing, Nanjing Medical University Affiliated Cancer Hospital, Cancer Institute of Jiangsu Province, Nanjing 210009, China

## Abstract

Long noncoding RNAs (lncRNAs) are known to regulate the development and progression of various cancers. However, few lncRNAs have been well characterized in lung adenocarcinoma (LUAD). Here, we identified the expression profile of lncRNAs and protein-coding genes via microarrays analysis of paired LUAD tissues and adjacent non-tumor tissues from five female non-smokes with LUAD. A total of 498 lncRNAs and 1691 protein-coding genes were differentially expressed between LUAD tissues and paired adjacent normal tissues. A novel lncRNA, LUAD transcript 1 (LUADT1), which is highly expressed in LUAD and correlates with T stage, was characterized. Both *in vitro* and *in vivo* data showed that LUADT1 knockdown significantly inhibited proliferation of LUAD cells and induced cell cycle arrest at the G0–G1 phase. Further analysis indicated that LUADT1 may regulate cell cycle progression by epigenetically inhibiting the expression of p27. RNA immunoprecipitation and chromatin immunoprecipitation assays confirmed that LUADT1 binds to SUZ12, a core component of polycomb repressive complex 2, and mediates the trimethylation of H3K27 at the promoter region of p27. The negative correlation between LUADT1 and p27 expression was confirmed in LUAD tissue samples. These data suggested that a set of lncRNAs and protein-coding genes were differentially expressed in LUAD. LUADT1 is an oncogenic lncRNA that regulates LUAD progression, suggesting that dysregulated lncRNAs may serve as key regulatory factors in LUAD progression.

Owing to smoking, air pollution and the aging of the population, the incidence and mortality rate of lung cancer is increasing rapidly.^1^ There is an obvious trend in recent years that the incidence of lung cancer caused by smoking is decreasing but that the incidence of lung adenocarcinoma (LUAD) in never smokers is growing.^2, 3^ According recent statistical data, the percentage of non-smoker lung cancer is approximately 25% of all cases of lung cancer, including 15% of all male and 53% of all female lung cancer patients. It has been well documented that LUAD in never smokers is remarkably different from that in smokers with aspect to etiology, clinical characteristics, genomic and transcriptomic factors.^4^ It is of paramount importance to identify the relationships between clinical symptoms and the molecular changes of LUAD among never smokers to develop new diagnostic and treatment strategies for LUAD and to improve the prognosis of diagnosed patients.

Long noncoding RNA (lncRNA) is a type of RNA molecules larger than 200 nucleotides that lacks protein-coding capacity.^5, 6^ Owing to their lack of reading frames, lncRNAs were originally considered as transcriptional noise. However, emerging evidence has demonstrated that lncRNAs have important roles in various biological and pathological processes, such as the immune response,^7^ differentiation,^8^ metabolism,^9^ and cancer development and progression.^10, 11, 12^ As an emerging paradigm of cancer research, many cancer-specific lncRNAs have been identified, a set of which have been validated as biomarkers for metastasis or prognosis, such as metastasis associated long antisense transcript 1 (MALAT-1),^13^ HOX transcript antisense RNA (HOTAIR)^14^ and colon cancer-associated transcript 2 (CCAT2). MALAT-1, as indicated by its name, is a lncRNA that is highly expressed in metastatic LUAD and associated with poor prognosis.^13, 15^ Currently, high-throughput technology such as RNA-sequencing and microarrays analysis has enable the characterization of lncRNA expression profile in biological processes^16, 17, 18^ and diseases.^19, 20, 21^

We have focused on lncRNA and reported a LUAD-specific lncRNA, CCAT2 that is significantly upregulated in LUAD but not in lung squamous cell cancer (LSCC).^22^ Here, we reported the protein-coding genes and lncRNAs expression profile of LUAD in female non-smokers characterized by microarrays and the identification of a novel lncRNA LUAD **t**ranscript 1 (LUADT1). The LUADT1 gene is located at chromosomal locus 6q24.3 and transcribes a 453nt transcript. By binding to SUZ12, a core component of the polycomb repressive complex 2 (PRC2), LUADT1 epigenetically suppressed p27 expression via histone modification. The silence of LUADT1 induced cell cycle arrest and significantly inhibited tumor growth both *in vivo* and *in vitro*.

## Results

### Expression profiles of lncRNAs and protein-coding genes among never smokers with LUAD

**Figure 1:**
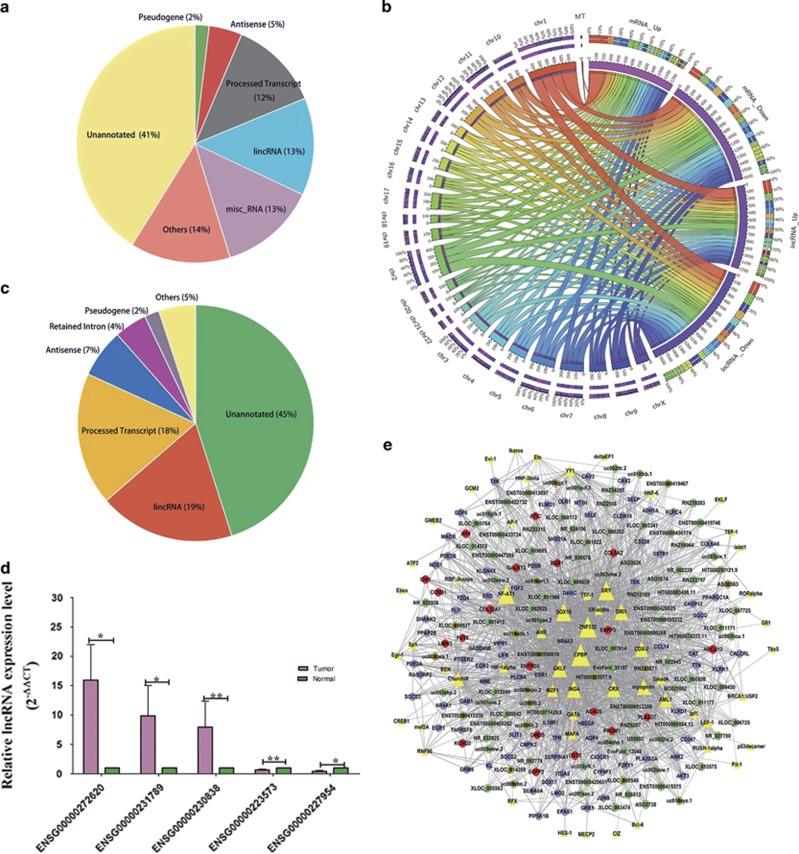
Expression profiling of lncRNAs and protein-coding genes via microarray analysis. (**a**) Biotypes of lncRNAs profiled. (**b**) Differentially expressed lncRNAs and protein-coding genes presented as a Circos plot. (**c**) Biotypes of differentially expressed lncRNAs. (**d**) The microarray results of five differentially expressed lncRNAs were selected for validation in 20 LUAD patients. ENSG00000272620: AFAP1-AS1; ENSG00000231789: PIK3CD-AS2; ENSG00000230838: AC093850.2; ENSG00000223573: TINCR; ENSG00000227954: TARID. *P<0.05, **P<0.01. (**e**) TF–lncRNA–protein-coding gene network; yellow: TFs; red: upregulated protein-coding genes; purple: downregulated protein-coding genes; green and red: upregulated lncRNAs; green and purple: downregulated lncRNAs. Error bars indicate means±S.E.M.

We used microarray analysis to characterize the expression profiles of lncRNAs and protein-coding genes in five never-smoking female LUAD patients. As shown in Figure 1a, most lncRNAs analyzed in the expression profile have not been annotated (Figure 1a). Filtered by *P-*value and fold change (*P*<0.05 and fold change >2.5 for lncRNAs; *P*<0.05 and fold change >3 for protein-coding genes), a total of 498 lncRNAs and 1691 protein-coding genes showed differential expression between the LUAD and paired adjacent normal tissues (Figures 1b and c). To validate microarray findings, five differentially expressed lncRNAs were selected and analyzed in a cohort of 20 female LUAD patients without smoking history (Supplementary Table S3) using qRT-PCR (Figure 1d). In agreement with microarray results, AFAP1-AS1, PIK3CD-AS2 and AC093850.2 were overexpressed and TINCR and TARID were downregulated in LUAD tissues (*P*<0.05 for all five lncRNAs). The top 100 differentially expressed lncRNAs and protein-coding genes are provided in Supplementary Table S4 and the microarray data have been uploaded to Gene Expression Omnibus database (accession number: GSE66654).

Transcription factors (TFs) have a central role in the regulation of gene expression. We constructed a TF–lncRNA–protein-coding gene network to illustrate how TFs modulate gene expression to identify the powerful TFs in LUAD.^23, 24^ As shown in Figure 1e, CPBP, ZNF333 and NF-AT1 were the three most dominant TFs, which affected the transcription of >140 genes. In addition, HIF1-alpha, SMAD4 and other oncogenic TFs also affect many genes. The co-expression networks were significantly different between the LUAD and normal lung tissues, suggesting that lncRNAs and protein-coding genes displayed different co-expression patterns between LUAD and normal tissues (Supplementary Figures S5 and S6). Gene ontology and KEGG pathway analyses were performed to identify the aberrant cellular functions and pathways (Supplementary Tables S7 and S8).

### The novel lncRNA LUADT1 is upregulated in LUAD

**Figure 2:**
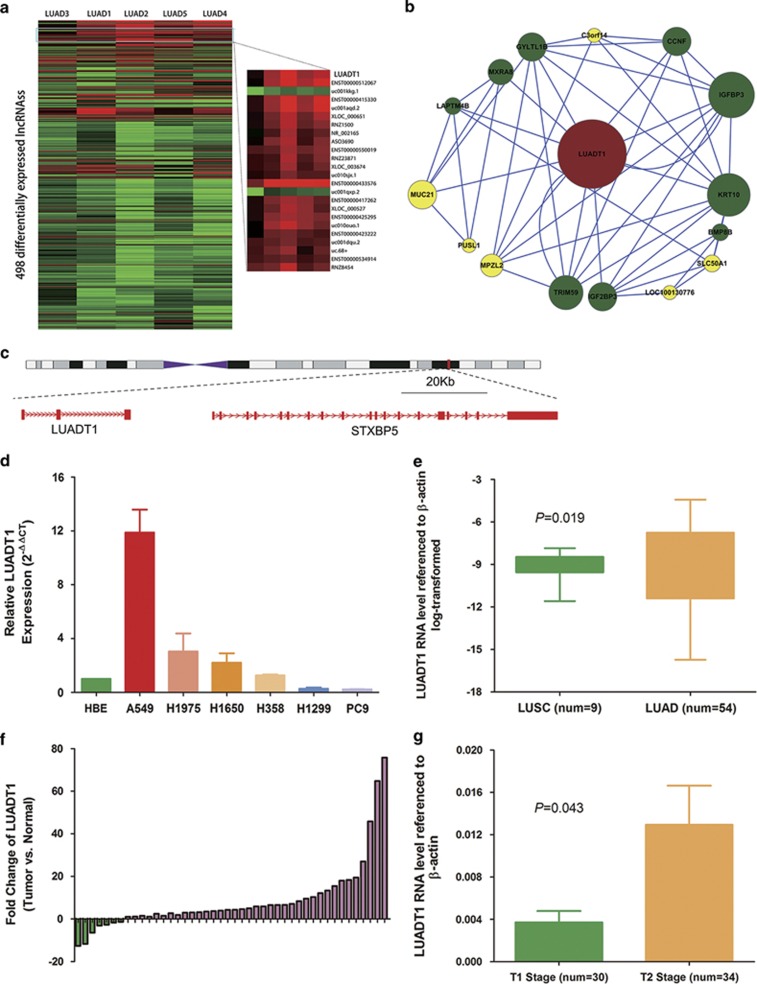
LUADT1 is upregulated in LUAD. (**a**) Hierarchical clustering showed that LUADT1 is significantly highly expressed; green: downregulated lncRNAs; red: upregulated lncRNAs. (**b**) Co-expression sub-network of LUADT1; green: genes associated with cell proliferation, drug resistance, tumor growth, invasion or prognosis; yellow: genes with unknown function. (**c**) Chromosomal location of LUADT1. (**d**) Expression of LUADT1 in LUAD cell lines. (**e**) LUADT1 is upregulated in NSCLC tissues compared with non-tumor tissues (8.34-fold, *P*<0.05). The LUADT1 expression level is higher in LUAD tissues than in LSCC tissues (**f**) and is higher in T2 stage tumors than in T1 stage tumors (**g**). Error bars indicate means±S.E.M.

Using hierarchical clustering,^25, 26^ a widely used data-mining tool, we identified a set of significantly differentially expressed lncRNAs (Figure 2a). As co-expression modules can represent biological pathways,^27^ we next analyzed sub-networks of these lncRNAs and their related protein-coding genes. Using this approach,^28^ we characterized a novel lncRNA, LUADT1, because its network included multiple protein-coding genes associated with tumor growth, invasion or prognosis (Figure 2b). The LUADT1 (ENSG00000196634) gene is located at chromosomal locus 6q24.3 locus (Figure 2c), which is within the lung cancer susceptibility locus 6q23-6q25,^29, 30^ and is transcribed as a 453nt lncRNA. The negative score of PhyloCSF^31^ (–12.7273, meaning that LUADT1 is 10^1.27273^ times more likely to be a noncoding sequence than a coding sequence), a comparative genomic method differentiating coding and noncoding RNA, and the lack of coding potential as determined by the coding potential assessment tool CPAT^32^ confirmed that LUADT1 is a noncoding RNA. Quantitative real-time PCR (qRT-PCR) was utilized to profile LUADT1 expression in lung cancer cell lines (Figure 2d). Compared with human bronchial epithelium (HBE), LUADT1 was remarkably overexpressed in the A549 cell line. In another cohort of 78 lung cancer patients, we investigated the expression level of LUADT1 and analyzed its clinical correlation (Table 1). As shown, LUADT1 was significantly overexpressed in lung tumor tissues compared with paired adjacent normal lung tissues, displaying an average difference of 8.34-fold (Figure 2e). The LUADT1 expression level was significantly higher in LUAD than in LSCC (*P*=0.019; Figure 2f) and correlated with the tumor stage (*P*=0.043; Figure 2g), but not with N stage or TNM stage.

### LUADT1 promotes cell proliferation *in vitro*

**Figure 3:**
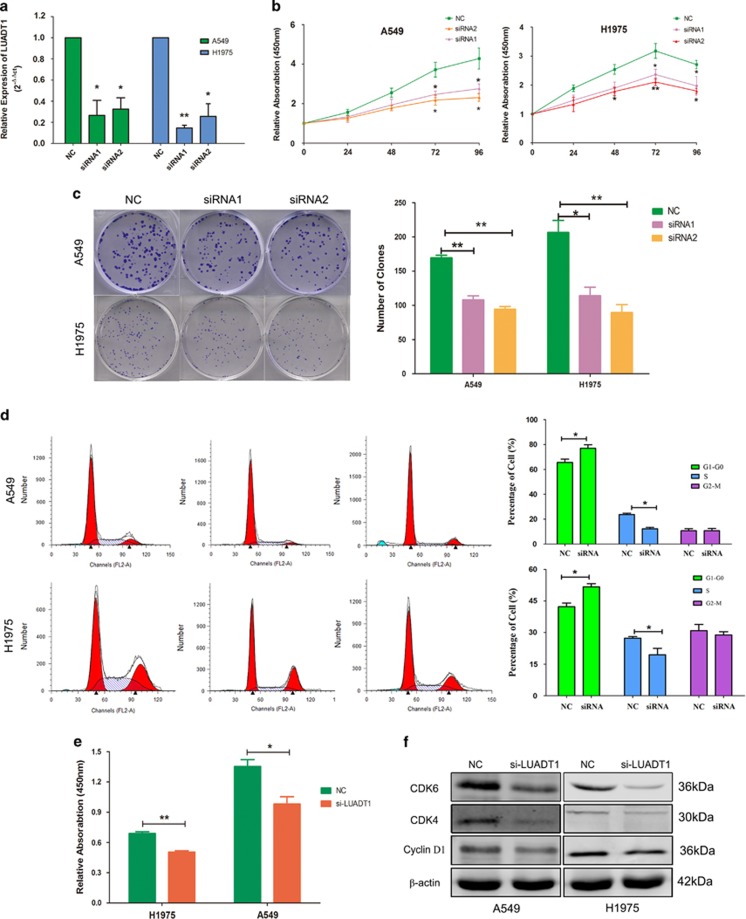
The silencing of LUADT1 inhibited LUAD cell proliferation *in vitro*. (**a**) Targeted siRNAs efficiently silenced LUADT1 in the A549 and H1975 cell lines. LUADT1 knockdown inhibited cell proliferation (CCK-8 assay, **b**) and clone formation **(c**) ability and induced cell cycle arrest at the G0–G1 stage (**d**) in A549 and H1975 cells. BrdU assay showed that siRNA-LUADT1 treatment significantly decreased relative absorbance at 450 nm (**e**). Western blot showed that cyclin D1, CDK4 and CDK6 were decreased after LUADT1 silence in A549 and H1975 cells (**f**). *P<0.05, **P<0.01. Error bars indicate means±S.E.M.

To evaluate the effects of LUADT1 on cell biological behavior, small interfering RNAs (siRNAs) were designed to silence LUADT1. The qRT-PCR results revealed that LAUDT1 was sufficiently silenced by siRNAs in two LUAD cell lines, A549 and H1975 (Figure 3a). Compared with the negative control (NC) siRNA, siRNAs targeting LUADT1 significantly inhibited cell proliferation ability in LUAD cell lines based on the CCK-8 assay (Figure 3b). Consistent with the results of the CCK-8 assay, colony formation ability was inhibited after LUADT1 knockdown as demonstrated by the decrease in the size and the number of colonies after siRNA-LUADT1 treatment (Figure 3c) compared with the NC treatment. We next examined whether the tumor cell cycle was affected after LUADT1 knockdown by Annexin V and propidium iodide double staining via FACS analysis. The results revealed that siRNA-LUADT1 treatment induced significant G0–G1 phase arrest and decreased the percentage of cells in the S phase (Figure 3d). BrdU assay also revealed that cell proliferation ability was significantly inhibited in both A549 and H1975 cells (Figure 3e). In consistence with BrdU and CCK-8 results, the expression of proliferation biomarkers, cyclin D1, cyclin-dependent kinase 4 (CDK4), and cyclin-dependent kinase 6 (CDK6) were decreased after LUADT1 silence (Figure 3f). Apoptosis was also analyzed after silence of LUADT1. Compared with NC, the rate of apoptotic cells was not affected by siRNA-LUADT1 treatment (Supplementary Figure S9). These data demonstrated that LUADT1 may promote LUAD cell proliferation, as well as cell cycle progression.

### The silence of LUADT1 suppressed tumor growth *in vivo*

**Figure 4:**
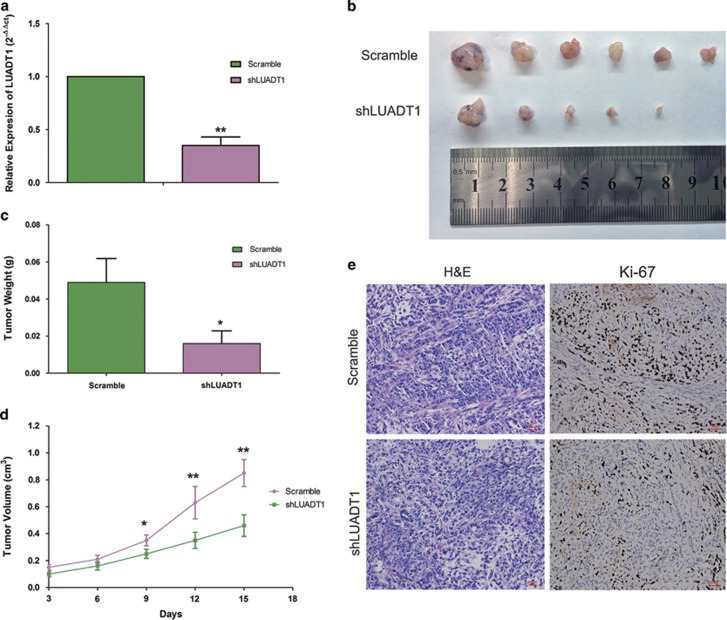
The silencing of LUADT1 inhibited LUAD growth *in vivo*. LUADT1-targeting or scrambled shRNA was transfected into A549 cells, and then, the cells were injected into nude mice. (**a**) LUADT1 expression was downregulated in the shLUADT1-transfected cell-derived xenograft tumors. The xenograft tumor weight (**c**) and volume (**b** and **d**) in the shLUADT1 group were significantly lower than those in the scrambled shRNA group. IHC staining was performed on xenograft tumors, and the Ki67 staining signal was weaker in the shLUADT1 group than in the scrambled shRNA group (**e**). *P<0.05, **P<0.01. Error bars indicate means±S.E.M.

To test whether LUADT1 regulates LUAD cell proliferation in *vivo*, we established xenograft tumor models in nude mice using A549 cells transfected with scrambled shRNA or shLUADT1. All nude mice developed xenograft tumors at the injection site, and the xenograft tumors were harvested 14 days after injection. qRT-PCR confirmed that the LUADT1 expression level was lower in the xenograft tumors derived from the shLUADT1-transfected cells (Figure 4a). As shown, the average tumor volume and weight in the shLUADT1 group was significantly lower than that in the control group (Figures 4b–d). IHC analysis revealed that tumors derived from shLUADT1-transfected cells showed weaker staining for Ki67, a cell proliferation marker,^33^ than those from scrambled shRNA-transfected cells (Figure 4e). These data demonstrated that LUADT1 may regulate tumor cell growth *in vivo*.

### LUADT1 suppresses p27 by binding to PRC2

**Figure 5:**
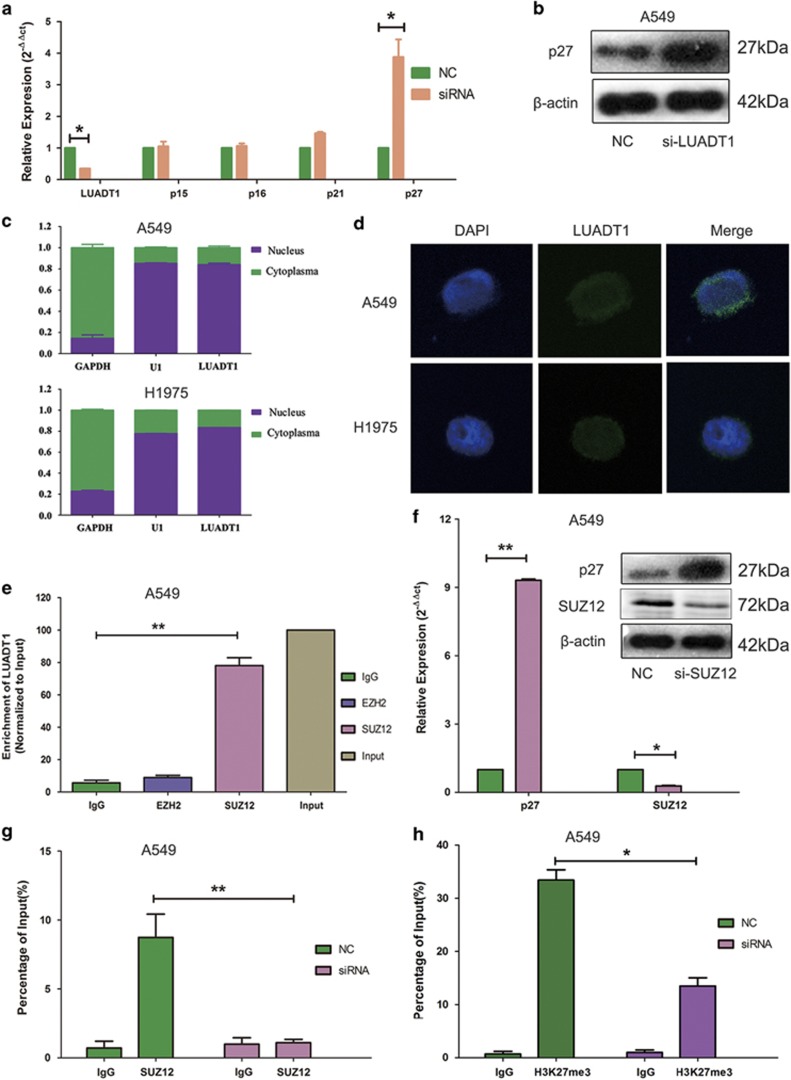
LUADT1 binds to SUZ12 to suppress p27 expression. The expression of cell cycle-related genes was analyzed after LUADT1 knockdown (**a**). Significant upregulation of p27 was observed and was confirmed by western blot (**b**). Cell fractionation assay revealed that LUADT1 is predominantly located in nucleus, and GAPDH and small nuclear RNA U1 were used as control genes of cytoplasm and nucleus (**c**). Fluorescence *in situ* hybridization assay demonstrated that most LUADT1 was located in nucleus (**d**). An RIP assay confirmed that LUADT1 binds to SUZ12, although the interaction between EZH2 and LUADT1 was not apparent **(e**). The silencing of SUZ12 decreased p27 expression at the mRNA and protein levels (**f**). The enrichment of SUZ12 and trimethylated H3K27 in the promoter region of p27 was detected via ChIP, and this enrichment was decreased after LUADT1 knockdown (**g** and **h**). *P<0.05, **P<0.01. Error bars indicate means±S.E.M.

As G0–G1 cell cycle arrest after LUADT1 knockdown was observed, we further explored the underlying mechanism using A549 as cell model. To identify the downstream targets of LUADT1, we first assessed the expression of the cyclin-dependent kinase inhibitor family via qRT-PCR (Figure 5a). Among these analyzed genes, we found that p27 (CDKN1B) was the most significantly upregulated gene after LUADT1 knockdown, and this result was confirmed by western blot (Figure 5b). p27 is an important tumor suppressor that is responsible for cell cycle control.^34^ Thus, we hypothesized that LUADT1 may promote LUAD cell proliferation via the suppression of p27 expression.

Subcellular location may provide clues regarding the molecular mechanism. Glyceraldehyde-3-phosphate dehydrogenase (GAPDH) and small nuclear RNA U1 (RNU1) were utilized as control of cytoplasm and nucleus, respectively. Compared with GAPDH and RNU1, LUADT1 was predominantly located in nucleus (Figure 5c). In addition, to visualize LUADT1 expression and subcellular location, fluorescence *in situ* hybridization (FISH) assay was performed. As shown (Figure 5d), most LUADT1 was localized in nucleus in both A549 and H1975. Khalil *et al.*^35^ have reported that approximately 20% of lncRNAs bind to PRC2, indicating that most lncRNAs exert their biological function by binding to RNA-binding proteins (RBPs), especially PRC2. Thus, we hypothesized that LUADT1 may regulate p27 expression by recruiting PRC2. PRC2 consists of three components, EZH2, SUZ12 and EED1. We first predicted the binding ability of LUADT1 to two key components, EZH2 and SUZ12, using an online algorithm, RPISeq.^36^ Using the EZH2–HOTAIR interaction pair as a positive control (interaction probability=0.75), we found that the SUZ12–LUADT1 interaction pair (interaction probability=0.7) had a higher score than the EZH2–LUADT1 interaction pair (interaction probability=0.45). These results were confirmed using another online tool, catRAPID.^37^ Next, we performed RNA immunoprecipitation (RIP) using antibodies against SUZ12 and EZH2 and observed a significant enrichment of LUADT1 using the SUZ12 antibody, but not the EZH2 antibody (Figure 5e), compared with the nonspecific IgG control antibody. Together, these data confirmed physical interaction between SUZ12 and LUADT1.

We further investigated the functional relevance of the interaction between SUZ12 and LUADT1. SUZ12 was first silenced using siRNA, and significant upregulation of p27 was observed (Figure 5f). Current evidence has demonstrated that the PRC2 complex is a negative regulator of transcription via histone modification, that is, the trimethylation of histone 3 lysine 27 (H3K27me3).^38^ Thus, it is very likely that LUADT1 suppresses p27 expression by recruiting the PRC2 complex to p27 promoter region, leading to trimethylation of H3K27 at this region. By performing chromatin immunoprecipitation (ChIP) experiments using antibodies against SUZ12 and H3K27me3, we detected the enrichment of SUZ12 and H3K27me3 in the promoter region of p27 (Figure 5g). After LUADT1 silencing using siRNA, SUZ12 enrichment in the promoter region of p27 was significantly decreased, and the occupancy of H3K27me3 in the p27 locus also decreased (Figure 5h).

**Figure 6:**
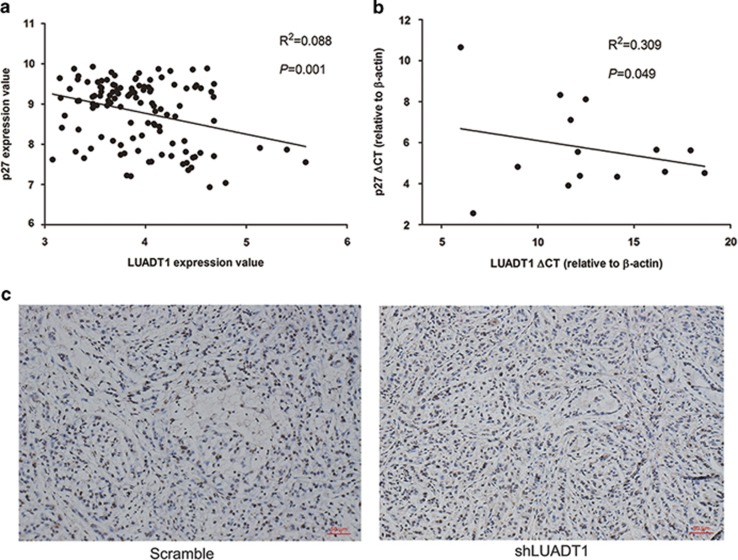
Negative correlation between LUADT1 and p27 expression. (**a**) The LUADT1 and p27 expression levels were negatively correlated in a lung cancer data set of 117 patients (GSE37138). This negative correlation was confirmed in our expression cohort via qRT-PCR (**b**). IHC staining of xenograft tumors showed that the p27 staining signal was stronger in the shLUADT1-transfected cell-derived xenograft tumors, in which LUADT1 expression was reduced (**c**)

Based on the above findings, we examined whether LUADT1 regulates p27 in lung cancer patients. For this purpose, we first analyzed our microarray results and found that the expression levels of p27 and LUADT1 were negatively correlated. Next, in a published microarray data series of 117 LUAD patients (GSE37138),^39^ we confirmed that the expression level of p27 negatively correlated with that of LUADT1 (Figure 6a). In the expression cohort of 20 LUAD patients, qRT-PCR results showed that the p27 mRNA level negatively correlated with the expression of LUADT1 (Figure 6b). Consistent with in these results, IHC analysis revealed that p27 staining was significantly stronger in the xenograft tumors derived from shLUADT1-transfected cells (Figure 6c).

Thus, these lines of evidence demonstrated that LUADT1 binds to SUZ12 and epigenetically inhibits p27 expression by mediating H3K27 trimethylation at the promoter region of p27.

## Discussion

Effective and individualized treatment of LUAD has not been well established. Understanding the gene expression profile and identifying the aberrantly expressed genes in LUAD may represent the crucial nodal points for the diagnosis and the therapeutic intervention of LUAD. Previous studies have demonstrated that lncRNAs have an important role in cancer by functioning as tumor suppressors^40, 41^ or oncogenes.^10, 28, 42^ For lung cancer, several dysregulated lncRNAs have been reported,^11, 13^ but these characterized lncRNAs are only the tip of iceberg, as most lncRNAs have not been investigated.

Based on microarray analysis, we determined the expression profiles of lncRNAs and protein-coding genes in five female non-smokers of LUAD and constructed an interaction network between lncRNAs and protein-coding genes, revealing the complex regulatory relationship between different types of genes. By bioinformatics methods, we identified a novel lncRNA, LUADT1. The expression level of LUADT1 was higher in LUAD than LSCC but we failed to find association between LUADT1 expression and smoking (Table 1). Experiments showed that LUADT1 promoted LUAD cell proliferation by epigenetic suppression of p27. Inhibition of LUADT1 significantly inhibited LUAD cell proliferation both *in vitro* and *in vivo*, suggesting LUADT1 could be a therapeutic target of LUAD.

In most cases, lncRNAs exert their function by binding to various RBPs, such as WDR5,^43^ GADD45A^41^ and hnRNPK.^44^ Among these RBPs, PRC2 has got most attention. PRC2 is a critical regulator of histone modification, which catalyzes the trimethylation of H3K27 to mediate gene silencing. Recent findings implicate that PRC2 is an important driver of tumor development and progression by suppressing various key genes, such as CDH1, DKKI and INK/ARF.^45^ DNA-binding factors are involved the recruitment of PRC2 to specific target genes. Increasing evidence has shown that in addition to proteins, many lncRNAs^10, 28^ physically associate with PRC2 and mediate H3K27 trimethylation at distinctive target loci. In this study, we demonstrated that LUADT1 binds to SUZ12 and epigenetically suppresses p27 expression.

p27 is a tumor suppressor that regulates cell cycle proliferation and is often downregulated in cancers. In lung cancer, low p27 expression is associated with poor prognosis.^46, 47^ We confirmed that the expression levels of p27 and LUADT1 were negatively correlated in LUAD patients. Moreover, in shLUADT1-transfected cell-derived xenograft tumors, p27 staining was lower than that in control cell-derived xenograft tumors. These data indicated LUADT1 is a robust negative regulator of p27. We also found that LUADT1 expression significantly positively correlated with T stage (Figure 2h). This result is consistent with the function of LUADT1 because LUADT1 may promote proliferation of LUAD; thus, a higher expression level of LUADT1 indicates a larger tumor size, that is, T stage.

Owing to the prognostic value of p27, the negative correlation between p27 and LUADT1 implies that LUADT1 may also be a prognostic marker for LUAD. However, owing to the limited follow-up information, the prognostic performance of LUADT1 has not been validated. It is highly possible that LUADT1 could regulate a set of other genes and RNA-sequencing or microarray analysis following LUADT1 knockdown may help to identify the downstream targets of LUADT1.

In this study, we identified a set of aberrantly expressed lncRNAs and protein-coding genes in LUAD. Our study paves the road for future investigations of biomarkers for LUAD and the comprehensive understanding of the molecular mechanisms by which lncRNAs affect LUAD.

## Materials and Methods

### Patients and tissue samples

This study was approved by the Ethics Committee of Cancer Institute of Jiangsu Province. Paired non-small cell lung cancer tissues and adjacent non-tumor tissues were obtained from patients who received surgical resection of NSCLC between 2012 and 2014 at the Department of Thoracic Surgery of Cancer Institute of Jiangsu Province, China. The pathological stage, grade and nodal status of all paired tumor and non-tumor tissues were confirmed by experienced pathologists. Clinical and pathological characteristics were also collected for each patient. Informed written consent was obtained from all patients included in this study.

### Microarray and computational analysis

The microarray experiment was performed by CapitalBio Corporation, Beijing, China. Expression profiling of lncRNAs and protein-coding genes was performed using the Agilent human lncRNA+mRNA array V.2.0 platform (Agilent Technologies, Santa Clara, CA, USA). The microarray data have been submitted to the Gene Expression Omnibus and the data could be accessed by the accession number, GSE66654. Five LUAD tissues corresponding non-tumor tissues (Supplementary Table 1) were used for microarray analysis. For TF–gene network, the sequences of differentially expressed genes were retrieved and analyzed with the Jemboss software (The European Molecular Biology Open Software Suite Team) to identify the relationships between genes and TFs. Next, a transcription TF–gene network was constructed based on the interactions between genes and TFs. We built lncRNA-protein-coding genes network to identify the interactions between protein-coding genes and lncRNA.^48^ For each gene–lncRNA, gene–gene or lncRNA–lncRNA pair, we calculated the Pearson correlation coefficient and selected the significantly correlated pairs to construct the network.^23^

### Cell lines and culture conditions

A549, H1975, H358, H1650 and H1299 cells were cultured in RPMI 1640 medium (KeyGEN, Nanjing, China) and Pc9 and HBE cells were cultured in DMEM medium, supplemented with 10% FBS (GIBCO-BRL, Invitrogen, Carlsbad, CA, USA), 100 U/ml penicillin and 100 mg/ml streptomycin (KeyGEN) in humidified air at 37 °C with 5% CO_2_.

### RNA extraction and qRT-PCR

The total RNA was extracted from tissues or cultured cells with TRIzol reagent (Invitrogen, Grand Island, NY, USA), according to the manufacturer's protocol. One microgram total RNA was reverse transcribed in a final volume of 20 *μ*l using PrimerScript RT Master Mix (Takara, Dalian, China; cat. no. RR036A). qRT-PCR was performed as previously described.^22^

### Subcellular fractionation location

The separation of the nuclear and cytosolic fractions was performed using the PARIS Kit (Life Technologies, Carlsbad, CA, USA) according to the manufacturer's instructions.

### Fluorescence *in situ* hybridization

Cells were fixed in 4% formaldehyde/5% acetic acid for 15 min followed by washes with PBS. The fixed cells were further treated with pepsin (1% in 10 mM HCl) and subsequent dehydration through 70%, 90% and 100% ethanol. The air-dried cells were subjected to incubation with 40 nM FISH probe in hybridization buffer (100 mg/ml dextran sulfate, 10% formamide in 2 × SSC) at 80 °C for 2 min. The hybridization was performed at 55 °C for 2 h and the slide was washed with 0.1 × SSC at 65 °C followed by dehydration through 70%, 90% and 100% ethanol. The air-dried slide was mounted with Prolong Gold Antifade Reagent with DAPI for detection. RNA FISH probe were designed and synthesized by Bogu Co., Ltd (Shanghai, China). Probe sequences are listed in Supplementary Table 2.

### Transfection of cell lines

Typically, LUAD cells were seeded at six-well plates and then transfected in the next day with specific siRNA (100 nM) or control siRNA (100 nM) using Lipofectamine RNAi MAX, according to the manufacturer's protocol (Invitrogen). The shRNA sequence of LUADT1 (5'-GATCCCCAGCAATCCTCTTACAGCAATTCAAGAGATTGCTGTAAGAGGATTGCTTTTTTGGAAA-3′) was cloned into pENTR/U6 vector. The primer sequences and siRNA sequences are summarized in Supplementary Table 2.

### Cell proliferation assay

A549 and H1975 cells were harvested 24 h post transfection by trypsinization. The Cell Counting Kit-8 assay was used to determine cell growth according to the manufacturer's instructions (KeyGEN). BrdU experiments were performed using a BrdU Cell Proliferation Assay Kit (Millipore, Billerica, MA, USA; cat. no. 2750) according to the manufacturer's instructions. The higher OD reading represents the higher BrdU concentration in the sample. The absorbance was measured at 450 nm with an ELx-800 Universal Microplate Reader(BioTek, Winooski, VT, USA). Each experiment was repeated at least three times independently.

### Flow cytometric analysis

For cell cycle analysis, cells were cultured with serum-free medium 24 h before transfection to induce cell cycle synchronization. Transfected cells were harvested after transfection by trypsinization. After the double staining with fluorescein isothiocyanate (FITC)-Annexin V and propidium iodide was done by the FITC Annexin V Apoptosis Detection Kit (BD Biosciences, San Jose, CA, USA) according to the manufacturer's recommendations. The cells were analyzed with a flow cytometry (FACScan; BD Biosciences) equipped with a Cell Quest software (BD Biosciences). Cells for cell cycle analysis were stained with propidium oxide by the Cycle TEST PLUS DNA Reagent Kit (BD Biosciences) following the protocol and analyzed by FACScan. The percentage of the cells in G0–G1, S and G2–M phase were counted and compared.

### Western blot assay

Cells were harvested, and protein was extracted from transfected cells and quantified as previously described^49^ using 12% or 4–20% polyacrylamide gradient SDS gel. Anti-*β*-actin and anti-SUZ12 were from Abcam (Hong Kong, China). Anti-p27, anti-cyclin D1, anti-CDK4 and anti-CDK6 were from Cell Signaling Technology (Boston, MA, USA).

### RNA immunoprecipitation

RIP experiments were performed using a Magna RIP RNA-Binding Protein Immunoprecipitation Kit (Millipore) according to the manufacturer's instructions. Antibodies of EZH2 and SUZ12 were from Abcam.

### ChIP assays

The ChIP assays were performed using EZ-CHIP KIT according to the manufacturer's instruction (Millipore). H3K27 antibody was from Millipore. The ChIP primer sequences were provided in Supplementary Table S2.

### Immunohistochemistry

Xenograft tumor tissue samples were immunostained for p27 and Ki67. Anti-Ki67 was from Santa Cruz Biotechnology (Dallas, TX, USA).

### Xenograft experiment

Transient transfection was performed in A549 cells with shLUADT1 or scrambled control sequence using Lipofectamine 2000 (Invitrogen). After 48 h of transfection, the cells were collected and injected into either side of the posterior flank of the same male BALB/c nude mouse. The tumor volumes and weights were measured every 2 days in the mice; the tumor volumes were measured as length × width^2^ × 0.5. Sixteen days after injection, the mice were killed, the tumor weights were measured and the tumors were collected for further analysis. The LUADT1 levels were determined by qRT-PCR.

### Statistical analysis

Student's *t*-test, one-way ANOVA analysis and Spearman test were performed to analyze the data using SPSS 18.0 software (Armonk, NY, USA). *P*<0.05 was considered statistically significant.

## Figures and Tables

**Table 1:** Correlation between LUADT1 expression level and clinical features in lung adenocarcinoma

**Characteristics**	**Number of patients**	**Percentage**	**Expression^a^**	***P*-values**
*Age (years)*				
<65	42	63.64%	0.00848	0.961
>65	24	36.36%	0.00827	
				
*Gender*				
Male	33	50%	0.01079	0.246
Female	33	50%	0.00603	
				
*Smoking*				
Yes	17	25.76%	0.00653	0.151
No	49	74.24%	0.01303	
				
*Drinking*				
Yes	6	9.09%	0.01688	0.191
No	60	90.91%	0.00756	
				
*Family history*				
Yes	7	10.61%	0.00176	0.265
No	59	89.39%	0.00920	
				
*Histology type*				
SCC	9	13.64%	0.00249	0.037^b^
AD	54	81.82%	0.00751	
Others	3	4.54%	0.00286	
				
*Tumor size*				
<3 cm	17	26.15%	0.00421	0.278
>3 cm	48	73.85%	0.00823	
				
*Differentiation*				
Low	5	7.94%	0.00120	0.553
Middle	40	63.49%	0.00667	
High	18	28.57%	0.00804	
				
*T stage*				
T1	30	46.86%	0.00372	0.043^b^
T2	34	53.14%	0.01036	
				
*Lymph node metastasis*				
Yes	21	31.82%	0.006615	0.845
No	45	68.18%	0.00729	
				
*TNM stage*				
I	39	59.09%	0.00825	0.774
II	9	13.64%	0.00449	
III	16	24.24%	0.00642	
IV	2	3.03%	0.00103	

a Expression level relative to *β*-actin.

b Significant association

## Abbreviations

LUAD: lung adenocarcinoma
RIP: RNA immunoprecipitation
H3K27: histone 3 lysine 27
ChIP: chromatin immunoprecipitation
LUADT1: lung adenocarcinoma transcript 1
PRC2: polycomb repressive complex 2
