# Supplementary material for: A novel lncRNA, LUADT1, promotes lung adenocarcinoma proliferation via the epigenetic suppression of p27
Source: Cell Death Dis. 2015 Aug 20;6(8):e1858–. doi: 10.1038/cddis.2015.203 (PMC4558496; doi:10.1038/cddis.2015.203)
Supplement: Supplementary Table S1 [file cddis2015203x1.doc]

**Supplementary Table 1.** Clinical characteristics of 5 lung adenocarcinoma patients used for lncRNA and protein-coding gene microarray

| ID | Gender | Smoking | Age(year) | T Stage | N Stage | M stage | TNM stage |
| --- | --- | --- | --- | --- | --- | --- | --- |
| 225409 | Female | Never | 54 | 1b | 0 | 0 | Ⅰa |
| 225301 | Female | Never | 60 | 1b | 0 | 0 | Ⅰa |
| 225569 | Female | Never | 50 | 1b | 0 | 0 | Ⅰa |
| 224215 | Female | Never | 56 | 1a | 2 | 0 | Ⅲa |
| 224469 | Female | Never | 54 | 2a | 2 | 0 | Ⅲa |
