# Supplementary material for: A novel lncRNA, LUADT1, promotes lung adenocarcinoma proliferation via the epigenetic suppression of p27
Source: Cell Death Dis. 2015 Aug 20;6(8):e1858–. doi: 10.1038/cddis.2015.203 (PMC4558496; doi:10.1038/cddis.2015.203)
Supplement: Supplementary Table S3 [file cddis2015203x3.doc]

Table S1. Clinical Features of the 20 lung adenocarcinoma patients

| Gender | Smoking Status | Age(yr) | TNM |
| --- | --- | --- | --- |
| Female | Never smoker | 69 | Ia |
| Female | Never smoker | 55 | Ia |
| Female | Never smoker | 57 | Ia |
| Female | Never smoker | 52 | Ia |
| Female | Never smoker | 64 | Ia |
| Female | Never smoker | 55 | Ia |
| Female | Never smoker | 70 | Ia |
| Female | Never smoker | 65 | Ia |
| Female | Never smoker | 60 | Ia |
| Female | Never smoker | 63 | Ia |
| Female | Never smoker | 48 | IIIa |
| Female | Never smoker | 58 | IIIa |
| Female | Never smoker | 69 | IIIa |
| Female | Never smoker | 52 | IIIa |
| Female | Never smoker | 71 | IIIa |
| Female | Never smoker | 57 | IIIa |
| Female | Never smoker | 68 | IIIa |
| Female | Never smoker | 62 | IIIa |
| Female | Never smoker | 60 | IIIa |
| Female | Never smoker | 67 | IIIa |
