# Supplementary figures and images for: A novel lncRNA, LUADT1, promotes lung adenocarcinoma proliferation via the epigenetic suppression of p27
Source: Cell Death Dis. 2015 Aug 20;6(8):e1858–. doi: 10.1038/cddis.2015.203 (PMC4558496; doi:10.1038/cddis.2015.203)

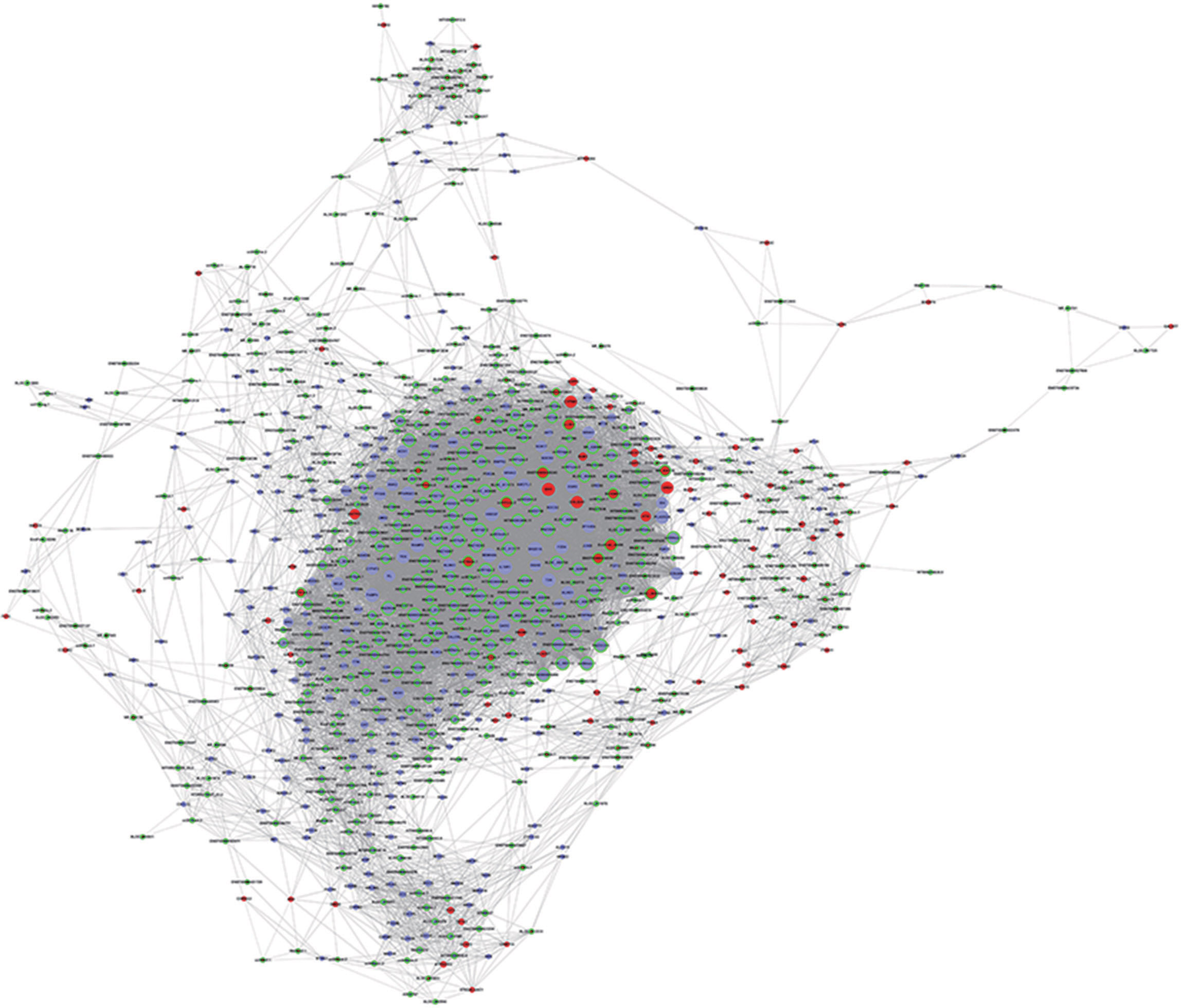

Supplement: Supplementary Figure S5 [file cddis2015203x5.tif]

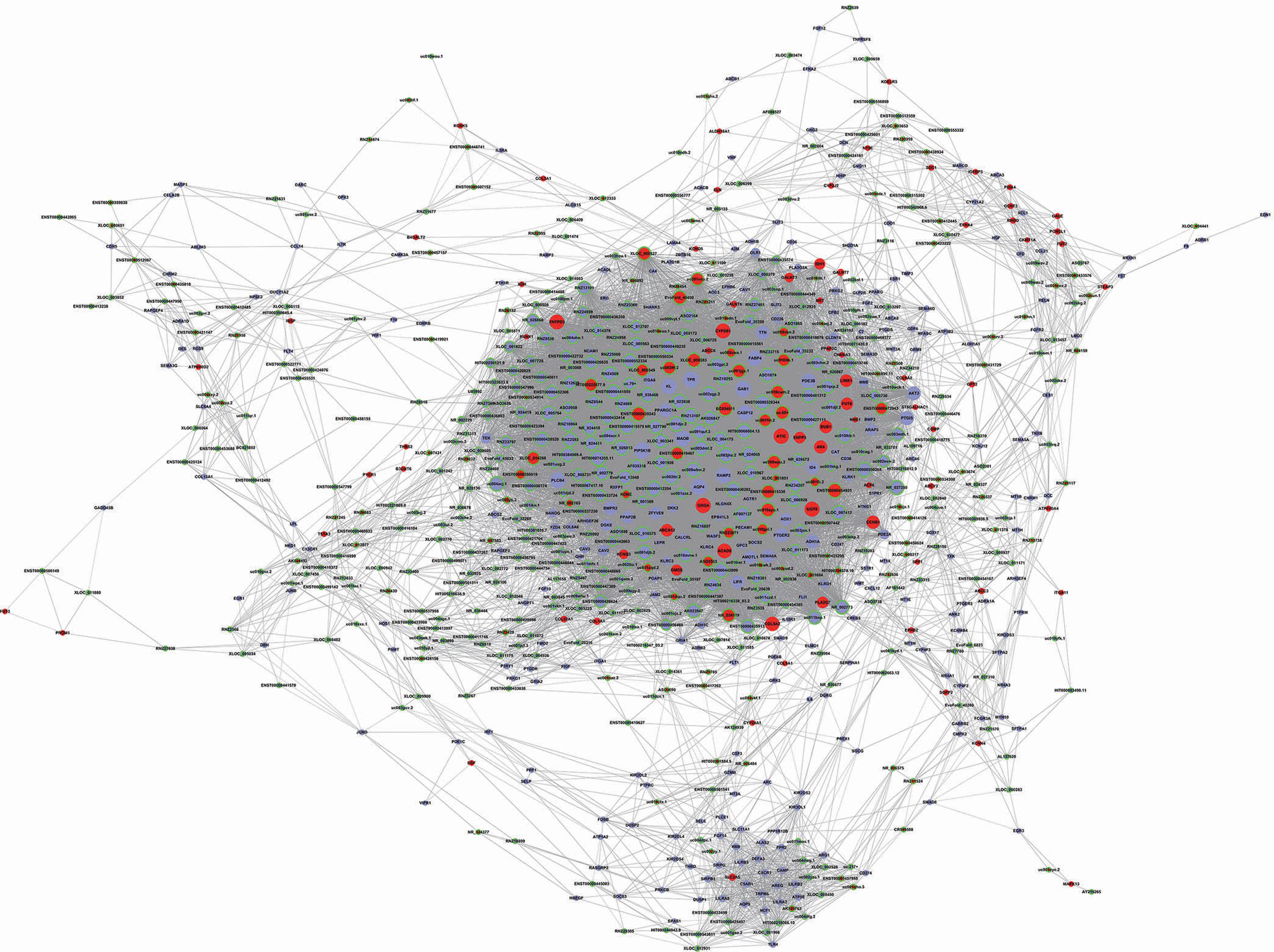

Supplement: Supplementary Figure S6 [file cddis2015203x6.tif]

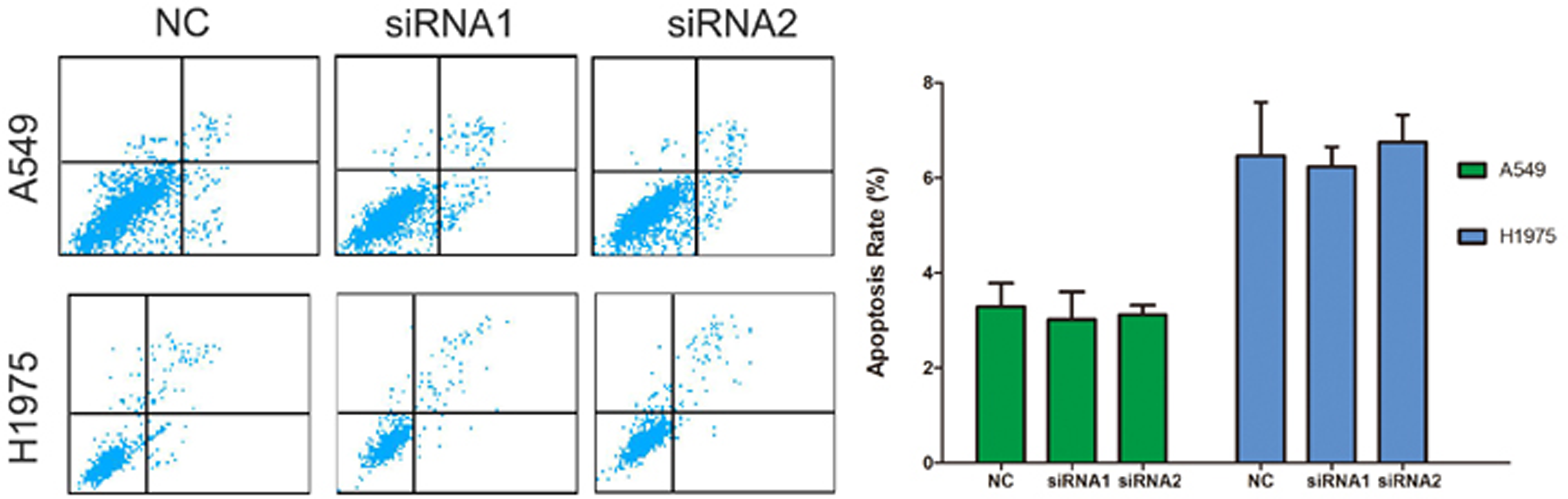

Supplement: Supplementary Figure S9 [file cddis2015203x9.tif]
