# Supplementary material for: A novel lncRNA, LUADT1, promotes lung adenocarcinoma proliferation via the epigenetic suppression of p27
Source: Cell Death Dis. 2015 Aug 20;6(8):e1858–. doi: 10.1038/cddis.2015.203 (PMC4558496; doi:10.1038/cddis.2015.203)
Supplement: Supplementary Information [file cddis2015203x10.doc]

**Supplementary Information**

**Supplementary Table 1**

Clinical characteristics of five lung adenocarcinoma patients in the microarray profiling analysis

**Supplementary Table 2**

Sequences of primers and siRNAs

**Supplementary Table 3**

Clinical characteristics of 20 lung adenocarcinoma patients for the validation of microarray results

**Supplementary Table 4**

The 100 most differentially expressed lncRNAs and protein-coding genes

**Supplementary Figure 5**

Co-expression network of lncRNA and protein-coding genes in lung adenocarcinoma tissues

**Supplementary Figure 6**

Co-expression network of lncRNA and protein-coding genes in non-tumor tissues

**Supplementary Table 7**

Significant altered Gene Ontology items

**Supplementary Table 8**

Significant altered KEGG pathways

Supplementary Figure 9

Silence of LUADT1 did not affect apoptosis. FACS analysis after staining showed that the rate of apoptotic cells did not change significantly after siRNA-LUADT1 treatment.
